# The effects of inorganic phosphate on muscle force development and energetics: challenges in modelling related to experimental uncertainties

**DOI:** 10.1007/s10974-019-09558-2

**Published:** 2019-10-16

**Authors:** Alf Månsson

**Affiliations:** Department of Chemistry and Biomedical Sciences, Faculty of Health and Life Sciences, Linnaeus University, Universitetskajen, 391 82 Kalmar, Sweden

**Keywords:** Myosin cross-bridges, Chemo-mechanical statistical model, Inorganic phosphate, Power, Force–velocity, Efficiency

## Abstract

Muscle force and power are developed by myosin cross-bridges, which cyclically attach to actin, undergo a force-generating transition and detach under turnover of ATP. The force-generating transition is intimately associated with release of inorganic phosphate (Pi) but the exact sequence of events in relation to the actual Pi release step is controversial. Details of this process are reflected in the relationships between [Pi] and the developed force and shortening velocity. In order to account for these relationships, models have proposed branched kinetic pathways or loose coupling between biochemical and force-generating transitions. A key hypothesis underlying the present study is that such complexities are not required to explain changes in the force–velocity relationship and ATP turnover rate with altered [Pi]. We therefore set out to test if models without branched kinetic paths and Pi-release occurring before the main force-generating transition can account for effects of varied [Pi] (0.1–25 mM). The models tested, one assuming either linear or non-linear cross-bridge elasticity, account well for critical aspects of muscle contraction at 0.5 mM Pi but their capacity to account for the maximum power output vary. We find that the models, within experimental uncertainties, account for the relationship between [Pi] and isometric force as well as between [Pi] and the velocity of shortening at low loads. However, in apparent contradiction with available experimental findings, the tested models produce an anomalous force–velocity relationship at elevated [Pi] and high loads with more than one possible velocity for a given load. Nevertheless, considering experimental uncertainties and effects of sarcomere non-uniformities, these discrepancies are insufficient to refute the tested models in favour of more complex alternatives.

**Electronic supplementary material:**

The online version of this article (10.1007/s10974-019-09558-2) contains supplementary material, which is available to authorized users.

## Introduction

The contractile machinery of striated muscle, i.e. skeletal muscle and heart, is arranged in ~ 2 µm long repetitive units, sarcomeres that are serially connected along ~ 1 µm wide myofibrils running the length of the muscle. The myofibrils are also connected in parallel over the muscle cross-section via cytoskeletal components. The main protein elements of the sarcomere are thick myosin containing filaments in the centre of each sarcomere and overlapping thin actin-containing filaments in the periphery. Muscle contraction is due billions of myosin II motor domains in the thick filaments forming cross-bridges with actin binding sites on the thin filaments in a cyclic process (Huxley 1974). Each cross-bridge cycle produces force and movement by synchronizing different steps in the turnover of ATP on the myosin active site with changes in actin affinity and with swing of an integral lever arm in the cross-bridge (see also Barclay et al. 2010; Batters et al. 2014; Månsson et al. 2015, 2018). The total developed muscle force results from summing the contributions from the chemo-mechanical cycles of all cross-bridge in all half-sarcomeres over the muscle cross-section. The muscle shortening velocity, on the other hand, is given by the speed of the thick versus thin filament sliding, multiplied by the number of half-sarcomeres in series. Due to the highly ordered serial and parallel arrangement, the mechanical and energetic properties of the muscle directly reflect the kinetic and mechanical properties of the ensemble of interacting myosin motors and thin actin-containing filaments in each given half-sarcomere. Because of the very large number of interacting myosin and actin molecules, statistical chemo-mechanical models are required to relate molecular properties to the experimental data. These models consider a very large ensemble of actin-myosin cross-bridges with a distribution of positions. After defining the kinetics, mechanics and coarse-grain structure based on independent experimental data, probabilities of different cross-bridge states under steady-state conditions are calculated by solving a system of ordinary differential equations in the state probabilities (Hill 1974; Huxley 1957). Combined information from solution biochemistry and single molecule mechanics, e.g. from optical tweezers based experiments, is sufficient to fully define the models including strain-dependence (Eisenberg et al. 1980; Hill 1974; Månsson et al. 2018), with minor exceptions regarding very fast strain-dependent rates. The latter are nevertheless obtained from independent experimental data (Månsson 2016). Observable variables such as muscle force, ATP turnover rate, etc. are derived by averaging over all cross-bridges. The first statistical chemo-mechanical model of the described type was that of Huxley (1957) which succeeded in accounting for key energetic aspects of muscle function. This includes the near hyperbolic shape of the relationship between an imposed constant load and the steady shortening velocity (the force–velocity relationship) (Hill 1938) and thereby the bell-shaped relationship between power and force. Additionally, the model accounted for the increased energy output (heat + work) during shortening compared to isometric contraction, the so called Fenn effect (1923). Following the pioneering work in Huxley (1957) and later in Huxley and Simmons (1971), a range of statistical cross-bridge models have been developed (e.g. Caremani et al. 2013; Duke 1999; Edman et al. 1997; Eisenberg and Greene 1980; Eisenberg and Hill 1978; Eisenberg et al. 1980; Julicher and Prost 1995; Månsson 2010a; Mijailovich et al. 2016, 2017; Offer and Ranatunga 2013; Piazzesi and Lombardi 1995; Smith 2014; Smith and Geeves 1995; Smith et al. 2008; Smith and Mijailovich 2008; Vilfan et al. 1999). They often rely on the theoretical formalism of Hill (1974) but have become increasingly complex with time to account for a wider range of more detailed experimental findings. To this end, recent models have also incorporated states and transitions with far from universal support in independent studies such as solution biochemistry and single molecule mechanics. This includes branched pathways or loose chemo-mechanical coupling (Caremani et al. 2013; Debold et al. 2013) slippage between sites (Caremani et al. 2013), cooperative phenomena (Huxley and Tideswell 1997; Julicher and Prost 1995), etc. In order to account for other findings such as effects of altered sarcomere length, it has also been found important to develop spatially explicit models where different aspects of the varying geometrical arrangement of individual cross-bridges is taken into account (Mijailovich et al. 2016; Smith et al. 2008; Tanner et al. 2007; Williams et al. 2010). Finally, the role of accessory proteins and mechanosensing (Linari et al. 2015) as well as emergent properties may be important to account for some phenomena particularly with varying levels of tension and during stretch (Campbell 2009; Marcucci and Reggiani 2016). The introduction of features such as branched pathways and loose coupling complicates model definition because these features have, to the best of my knowledge, no independent support from solution biochemistry and single molecule mechanics. The parameter values must therefore be assigned by a fitting procedure where they are adjusted to improve the fit to muscle mechanic and energetic data, i.e. the results that are actually modelled. This is opposed to the present approach where states and parameter values are assigned based on independent experimental data (primarily solution biochemistry and single molecule mechanics). Further adjustments by fitting procedures are generally not made (unless explicitly stated in some cases) to improve fits to muscle mechanical and energetic data.

Despite the perceived need for increasingly complex models, it was found recently (Månsson 2016, 2019; Månsson et al. 2018, 2019; Rahman et al. 2018) that assignment of parameter values as described in the preceding paragraph, allow models to account surprisingly well for a range of critical aspects of muscle contraction. Both isometric contraction and shortening contractions are thus accounted for without the need to invoke cooperative transitions (Huxley and Tideswell 1997; Månsson 2010a), inter-site slippage (Caremani et al. 2013), effects of accessory proteins (Tanner et al. 2007), branched pathways (Caremani et al. 2013; Debold et al. 2013), 3D order (Mijailovich et al. 2016) or emergent phenomena due to higher order sarcomere behaviour (Campbell et al. 2011). The models (Månsson 2016, 2019; Månsson et al. 2019; Rahman et al. 2018) without these complexities are here denoted “simple models”. These models have generally assumed just one myosin binding site per target zone spaced at 36 nm intervals along the actin filament. However, expanding the number of sites per target zone from one to three did not appreciably change the predictions if appropriate scaling was implemented (e.g. of force and ATP turnover rate) (Månsson 2019). The “simple” models (Månsson 2016, 2019; Månsson et al. 2019; Rahman et al. 2018) not only account for physiological muscle properties using model parameters derived from the bottom up in experiments on isolated actin and myosin. The models, particularly that in (Rahman et al. 2018), also account for central aspects of the mechanism for release of inorganic phosphate (Pi) and its relation to force-generation. However, the long-term controversy regarding this process (cf. Månsson et al. 2015; Stehle and Tesi 2017 and references therein), has not yet been resolved. Particularly, it has not been tested if the “simple” models (Månsson 2016, 2019; Månsson et al. 2019; Rahman et al. 2018) can account for effects of varied [Pi] on the force–velocity relationship and muscle energetics. Such tests are important because details of the Pi-release process in relation to force-generation are reflected in effects of varied [Pi] on force and movement (Debold et al. 2013; Pate and Cooke 1989; Smith 2014; Stehle and Tesi 2017).

Here, two simple models (Månsson 2019; Rahman et al. 2018) as defined above, are used to test the hypothesis that key contractile and energetic effects of altered [Pi] can be accounted for without introducing branched pathways or loose coupling between force-generation and Pi-release. Furthermore, the models rely on structural data (Llinas et al. 2015) and theoretical arguments (Smith 2014) in assuming that Pi-release occurs before force-generation (the power-stroke), an idea that is not universally accepted (Muretta et al. 2013; Trivedi et al. 2015; Woody et al. 2019) (reviewed in Månsson et al. 2015). The results demonstrate that the model faces challenges in accounting for some effects of altered [Pi]. However, consideration of the model predictions in relation to experimental complexities, suggests that the challenges are not sufficiently severe to justify refutation of the models in favour of more complex alternatives. Further studies are needed to resolve the conflicts between experimental findings and predictions and some suitable tests are suggested below.

## Methods

**Fig. 1:**
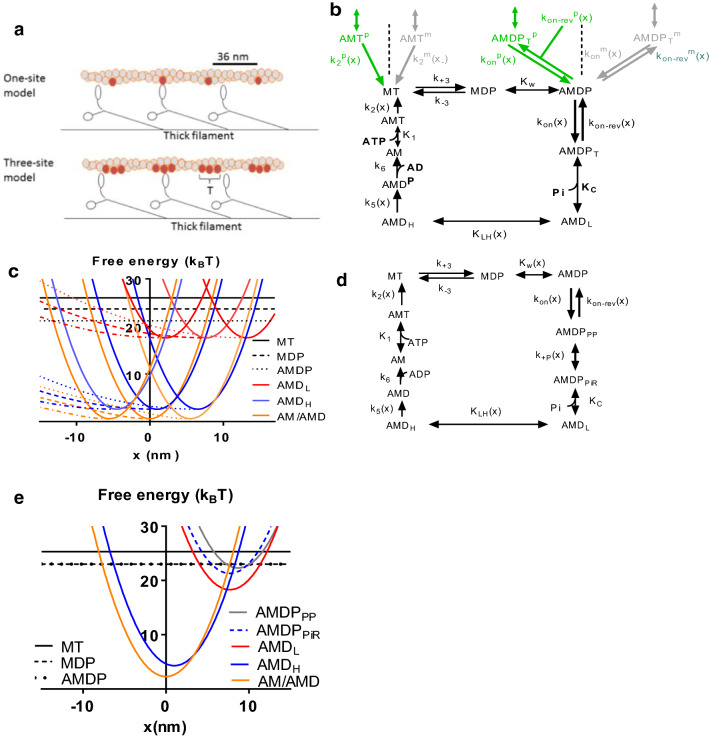
Models used. **a** Schematic illustration of model geometry for target zones (T) on actin with 1 (10:1 model) and 3 (9:3 model) sites. **b** Kinetic scheme for 1 site in the 3-site model with 9 states (9:3 model). The myosin head (M) is either attached to actin (A) or not. Except for in the “rigor” state (AM), the heads have ATP (T), ADP (D) and/or inorganic phosphate (P, Pi) at the active site. The AMDP_T_ state is a transient intermediate under physiological conditions. Rate constants are written generically as k_i_ and equilibrium constants as K_i_. An argument (x) indicates strain dependence of the “constant”. The green and grey arrows refer to transitions to the states in green and grey font, respectively at the neighbouring sites along actin (superscript “p” and “m” towards the plus end and minus end of actin, respectively). **c** Free energy diagrams for the 9:3 model plotted versus variable x which is the distance of the myosin head from the position where the free energy in the AM state at the central site attains its minimum. The full and dashed coloured lines represent linear and non-linear cross-bridge elasticity, respectively. **d** Kinetic scheme for the 1-site model with 10 states (10:1 model). Similar terminology as in **a**. However, the AMDP_PP_ (pre-power stroke state) and the AMDP_PiR_ (Pi-release state) states are unique to the 10:1 model. **e** Free energy diagrams for the 10:1 model plotted versus variable x. Note, smaller free energy drop from state MT to state AM/AMD than in 9:3 model, primarily because of lower actomyosin affinity in the AMDP state in the 10:1 model (corresponding to 2.5 k_B_T difference). Note, for **b**–**e**, the AM and the AMD states in the kinetic schemes (**b**, **d**) are assumed to be structurally and energetically equivalent, justifying that they are lumped together into one state in the free energy diagrams (**c**, **e**). (Color figure online)

**Fig. 2:**
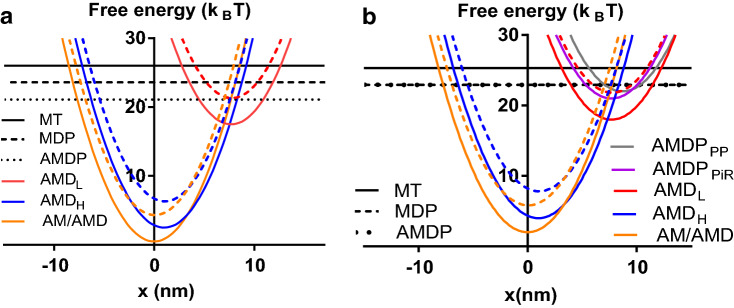
Effects of increase from 0.5 to 25 mM Pi on free energy diagrams. **a** Central site in the 9:3 model. Comparison of free energies of different strongly attached states at 25 mM Pi (dashed coloured lines) and 0.5 mM Pi (full coloured lines). **b** 10:1 Model. Comparison of free energies of different strongly attached states at 25 mM Pi (dashed coloured lines) and 0.5 mM Pi (full coloured lines). Both in **a** and **b** the absolute values of the free energies at 25 mM Pi are shifted upwards (to compensate for reduced free energy of ATP at increased [Pi]) so that the free energies of the MTP, MDP and the AMDP state (black lines) coincide at 0.5 mM and 25 mM Pi in both plots and also those of the AMDP_PP_ and the AMDP_PiR_ in **b**. (Color figure online)

The models used as a basis for the present simulations are those of Rahman et al. (2018) and Månsson (2019) (see also Månsson 2016) as further motivated in the “Discussion” section. The model of Månsson (2019) with 9 states per site is readily reduced to 6 states in the calculations (see SI). This follows from the transient intermediate states AMDP_T_ and AMT that are not explicitly considered and the states AM and AMD that are in rapid equilibrium and lumped into one AM/AMD state. Nevertheless, this model (Fig. 1a, b, c) is denoted “Model 9:3” due to 9 original states per site and 3 sites per target zone. The model of Rahman et al. (2018) with 10 states is implemented without explicitly considering the transient AMT state and by lumping together the AM and the AMD state into an AM/AMD state. Nevertheless, because of 10 states in the original model and 1 assumed site per target zone this model is denoted model 10:1 below (Fig. 1a, d, e). Importantly, all the states and inter-state transitions included in the models find general support in biochemical data (cf. Houdusse and Sweeney 2016; Månsson et al. 2015, 2019; Smith 2014). Furthermore, as specified elsewhere in this paper, the model parameter values (rate functions, cross-bridge stiffness, power-stroke distances, etc.) are all defined based on independent data, primarily derived in solution biochemistry and single molecule mechanics studies (see also “Model” section and Tables S1, S2 in the Supporting Information). The effects of an increase in [Pi] from 0.5 mM to 25 mM on the free energy diagrams of models 9:3 and 10:1 are depicted in Fig. 2. The implementation of both models, including methods for numerical solution of differential equations, is described in the Supporting Information. Further details can be found in the original papers (Rahman et al. 2018; Månsson 2019) (see also Månsson 2016).

### Experimental data from the literature

Experimental data from the literature were derived from copied figures in cited papers (pdf-documents) and measurements were made using ImageJ (Schneider et al. 2012).

## Results

**Fig. 3:**
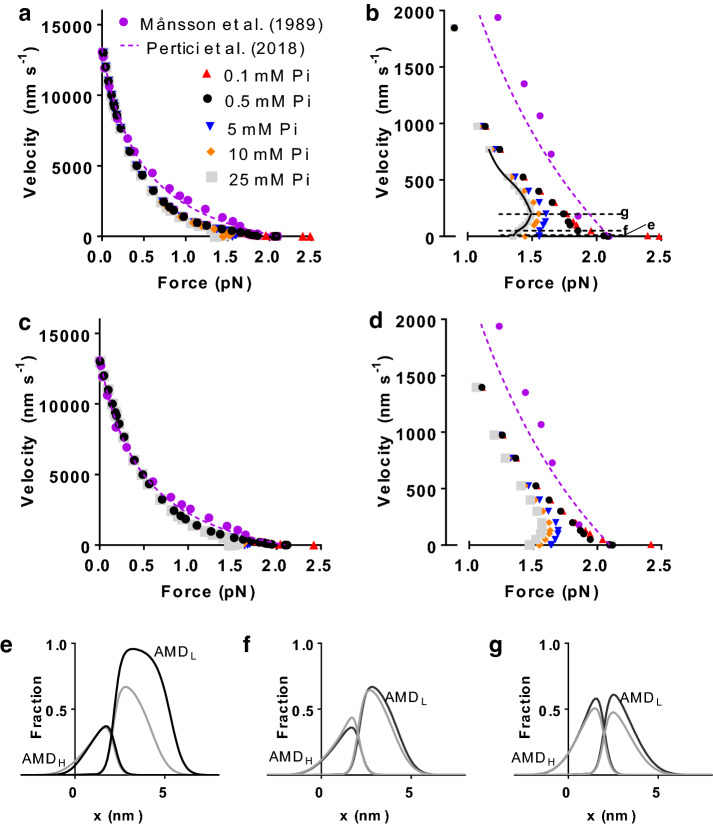
Force–velocity relationships at different [Pi]. Model with 9 states and 3 sites (9:3-model). **a** Model data for different [Pi] assuming linear cross-bridge elasticity. Comparison to experimental data from living fast mouse muscle (Månsson et al. 1989) and from actin–myosin ensembles in vitro (Pertici et al. 2018) under non-fatiguing conditions with expected low [Pi] (< 1 mM). **b** Details of the high-force, low-velocity region in **a**. Horizontal dotted lines “e”–“g” refer to cross-bridge distributions in **e**–**g**. Curved full line: a fit used in analysis below (see Fig. 5). **c** Similar analysis as in **a** but assuming non-linear cross-bridge elasticity. **d** Details of the high-force, low-velocity region in **c**. For ease of comparison the experimental force–velocity data are scaled to the maximum velocity predicted by the model. Furthermore as motivated in the text the experimental maximum isometric force is scaled to the maximum isometric force predicted by the model for [Pi] = 0.5 mM Pi. **e** Population of low-force (AMD_L_) and high-force (AMD_H_) states as function of strain-parameter x in isometric contraction (labelled “e” in **b**) at 0.5 mM (black) and 25 mM (grey) Pi. **f** Similar data as in **e** but at a shortening velocity of 50 nm/s (velocity indicated by line “f” in **b**). **g** Similar data as in **e** but at a shortening velocity of 200 nm/s (velocity indicated by line “g” in **b**)

As demonstrated previously (Månsson 2016, 2019), the 9:3 model assuming linear cross-bridge elasticity, accounts well both for the maximum velocity of shortening (V_0_, experimental range: 12,000–18,000 nm/s Asmussen et al. 1994; Månsson et al. 1989; Ranatunga 1984) and the shape of the force–velocity relationship. However, the predicted velocity is somewhat low at intermediate loads (giving low maximal power output) compared to experimental data. The predicted maximum isometric force (2.1 pN/myosin head) is lower than that estimated from isolated living single cells of mouse skeletal muscle (2.7 pN/myosin head^1^ Westerblad et al. 1997) at similar temperature (see discussion about the discrepancy in Månsson 2019). However, the predicted isometric force is appreciably higher than in the experimental data for whole muscle in Fig. 3 (1.0 pN/myosin head).^2^ We attribute the latter difference to overestimation of the myofibrillar cross-sectional area in the experiment, due to the presence of extracellular space that was not explicitly taken into account. Moreover, there may also be contribution towards lower force in the whole muscle due to oblique insertion of several muscle fibres into the tendons. For this reason the experimental data in all figures below are scaled to the maximum force simulated by the tested model at 0.5 mM Pi.

**Fig. 4:**
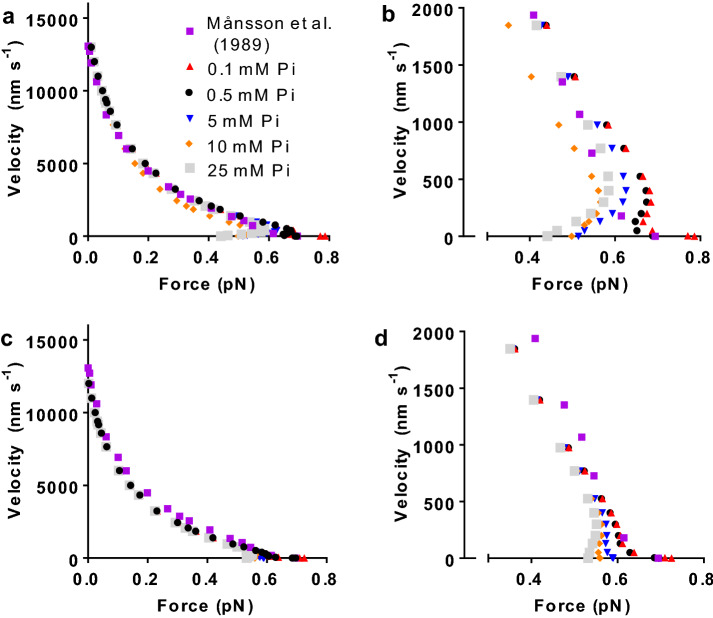
Force–velocity relationship at different [Pi] using model with 10 states and 1 site (10:1-model) and the assumption of linear cross-bridge elasticity throughout. **a** Original model of Rahman et al. (2018) with minor modification (Table S2). **b** Same data as in a, but in the high-force, low-velocity range only. **c** Model of Rahman et al. (2018) but with shift of x-positions for free energy minima of states AMDP_PP_ and AMDP_PiR_ from 8.7 to 7.2 nm and from 7.7 to 6.9 nm, respectively compared to original model. **d** Same data as in **c**, but in high-force, low-velocity range only. Experimental data from Månsson et al. (1989) same as in Fig. 3

Based on considerations in the “Discussion” section, the decision was made to investigate effects of altered [Pi] using the 9:3 model on the assumption of both linear and non-linear cross-bridge elasticity and the 10:1 model with linear elasticity only. Major differences between these models is that the maximum power output for the parameter values used (taken from independent experimental data) is lowest for the 9:3 model with linear elasticity (Fig. 3a, b), somewhat higher for the 9:3 model with non-linear cross-bridge elasticity (Fig. 3c, d), and highest (and in best agreement with the experimental data) for the 10:1 model (Fig. 4). All models account to varying degree for the non-hyperbolic shape of the force–velocity relationship at high loads (Devrome and MacIntosh 2007; Edman 1988) at close to physiological [Pi] (here taken as 0.5 mM).

The simulations using all tested models (Figs. 3, 4) show that varied [Pi] in the range 0.1–25 mM has negligible effects on the force–velocity relationship at velocities > ~10% V_0_ [velocity > ~ 700 nm/half-sarcomere/s (nm/s in the following)]. This lack of effect applies both to the shape of the relationship and the absolute velocity value for a given force in general agreement with experimental results at 0 and 10 mM added Pi (Caremani et al. 2013). Accordingly, the maximum velocity of shortening is also negligibly affected by altered [Pi] as previously found in experiments at neutral pH (Caremani et al. 2013; Cooke and Pate 1985; Debold et al. 2011). On the other hand, the maximum isometric force is appreciably reduced by increased [Pi] both in the 9:3 and the 10:1 model. For an increase of [Pi] in the range 0.5 mM to 25 mM modelled for conditions at 30 °C the reduction was 46% (linear elasticity) and 32% (non-linear elasticity) for the 9:3 model and 25 and 44% for the different versions of the 10:1 model (Fig. 4a, b vs. c, d; see also Fig. 6). For all model versions tested, increased [Pi] caused altered shape of the force–velocity relationship at velocities < ~10% V_0_. Interestingly this also seems to apply for experimental data but, to the best of my knowledge, this has only been tested (Caremani et al. 2013) for 10 mM added Pi. Moreover, also in the latter case, there are too few data points in the high-force range in the experiments (Caremani et al. 2013) to reveal the detailed effects of increased [Pi]. In the simulations, for all models tested here (with linear and non-linear elasticity), the force–velocity relationship at [Pi] > ~ 5 mM exhibits anomalous shape (cf. Julicher and Prost 1995; Vilfan et al. 1999) in the high-force range with more than one constant velocity for a given load. This effect becomes more accentuated the higher the [Pi] tested (up to 25 mM) and for the original version of the 10:1 model (Rahman et al. 2018) the effect is present even at normal physiological [Pi] (< 1 mM). A change to non-linear cross-bridge elasticity does not noticeably modify the effects of altered Pi on the force–velocity relationship in the 9:3 model (Fig. 3c, d).

The appearance of an anomalous force–velocity relationship upon increased [Pi] originates in the effects of increased [Pi] on the free energy diagrams (Fig. 2) and associated changes in transition rate constants and cross-bridge distributions. Critical effects on the cross-bridge distributions of increased [Pi] were analysed for the 9:3 model with linear cross-bridge elasticity in Fig. 3e–g. Here, cross-bridge distributions are depicted for 0.5 mM and 25 mM Pi at three different velocities in the high-force range as indicated in Fig. 3b. Under normal physiological conditions (0.5 mM Pi, black curves in Fig. 3e–g), going from isometric contraction (Fig. 3e) to very slow shortening (50 nm/s, Fig. 3f), the pre-power-stroke AMD_L_ state is markedly depleted at high strains (x > ~ 5 nm, where attachment rate is slowest). This effect is the basis for the appreciable reduction in force in the model when going from e to f in Fig. 3b. When the shortening velocity increases further, the post-power stroke AMD_H_ state becomes increasingly populated which tends to increase force. However, as the latter tendency is offset by further reduced population of cross-bridges with high strain in the AMD_L_ state there is no net increase in force upon further increased shortening velocity at low [Pi] but rather a lowered slope (change in velocity/change in force) of the velocity versus force plot [between f (50 nm/s) and g (200 nm/s) in Fig. 3b]. The described changes in the cross-bridge distributions between the velocities at e and f in Fig. 3b at 0.5 mM Pi are the basis for the non-hyperbolic force–velocity relationship (Edman et al. 1997) in the present model. In principle, similar mechanisms operate at 25 mM Pi as at 0.5 mM Pi. However, at 25 mM Pi, the Pi-induced reduction in actomyosin affinity in the AMD_L_ state depletes high-strain cross-bridges (x > ~ 5 nm, grey line in Fig. 3e) in the AMD_L_ state already in isometric contraction. Therefore the mechanism, with loss of AMD_L_ cross-bridges at high strain, that underlies the reduction in force when going from isometric contraction to slow shortening (50 nm/s) at low [Pi] is lost at high [Pi] (cf. grey lines in Fig. 3e, f). At high [Pi], this effect is completely overpowered by the increase in force attributed to increased population of the AMD_H_ state, explaining the higher force in the model at a velocity of 200 nm/s (point g in Fig. 3b) than during isometric contraction.

**Fig. 5:**
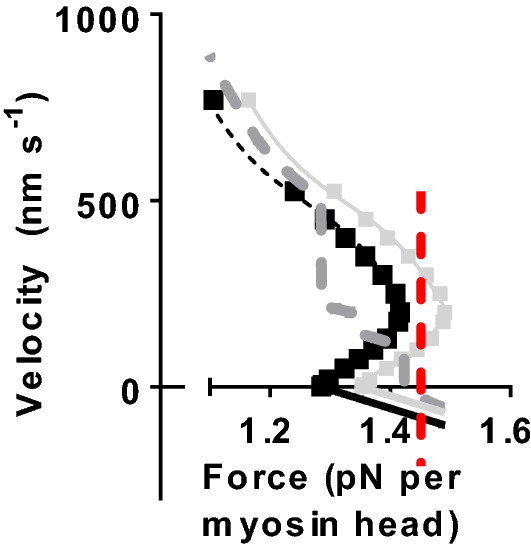
Anomalous force–velocity relationship in relation to sarcomere-non-uniformities. The grey squares and the full curved grey line are reproduced from the model in Fig. 3b. The black squares and the dashed black line represent the same data shifted towards lower maximum force to simulate lower force-producing capacity without changes in the shape of the force–velocity relationship. Black and grey sloped straight lines at negative velocity: tentative force velocity relationship during lengthening with higher absolute value of the derivative of force against velocity than during the shortening part of the force–velocity relationship (Edman 1988). Dashed dark grey line: average force–velocity relationship from two half-sarcomeres in series, derived as described in the text. Dashed vertical red line: a given load (force) level illustrating that three different constant velocities are possible for the strong half-sarcomere at this load. See text for details of the analysis. (Color figure online)

Small changes of the model parameter values within experimental uncertainties reduced the tendency for an anomalous force–velocity relationship at high [Pi] (Fig. S1), consistent with a coupled uncertainty and sensitivity analysis applied previously to the 10:1 model (Rahman et al. 2018). Thus reduced cross-bridge stiffness and/or small shifts in the positions of minima of the free energy in the pre-power stroke states (consistent with changes in the power-stroke distance) significantly reduced the tendency for an anomalous relationship. The former effect is consistent with the uncertainty in the cross-bridge stiffness in the range 1.7–3 pN/nm and the latter change is consistent with the uncertainty of the power-stroke distance in the range 7–10 nm (reviewed in Månsson et al. 2018). Related to the latter uncertainty and the uncertainty with regard to the amplitude of the first small structural change before phosphate release (Llinas et al. 2015; Rahman et al. 2018) the parameter values x_1_ and x_11_ were reduced from 8.7 to 7.2 nm for x_1_ and from 7.7 to 6.9 nm for x_11_ in the 10:1 model in Fig. 4c, d. Interestingly, these minor modifications tend to move the force–velocity relationship away from anomalous behaviour, particularly at low [Pi] (Fig. 4c, d). This example points to the possibility that minor changes in model parameters [within experimental uncertainties (± 25%) Rahman et al. 2018] could potentially eliminate the anomalous force–velocity relation at high loads. However, considering all possible combinations of such minor changes it is not meaningful to pursue this path in detail. This is particularly true because it seems likely that an anomalous force–velocity relationship within a half-sarcomere may go undetected in experiments. This follows because even length clamped segments of muscle fibres usually contain a very large number of sarcomeres. Now, assume that different half-sarcomeres differ in force-generating capacity (e.g. due to different degrees of overlap or other factors) as demonstrated previously (Edman and Flitney 1982; Edman and Reggiani 1984b; Edman et al. 1985; Gordon et al. 1966; Poggesi et al. 2005; Stehle 2017). Then, if the load on the segment is clamped (Caremani et al. 2008; Edman 1988; Edman and Curtin 2001) to a given level in the high-force region and if the force–velocity relationship is anomalous, each half-sarcomere may elongate or shorten at either of up to three constant velocities (cf. intersections of dashed vertical line in Fig. 5 with data indicated by light grey symbols). If there are two half-sarcomeres in series and if these have different force-generating capacity, then up to 3 × 2 = 6 different constant velocities are possible for the entire segment. Assuming random jumps between the different stable velocities for each half-sarcomere it seems relevant to use the average of all, up to 6, different possible velocities of the segment as that which would be measured in an experiment. Under the conditions assumed in Fig. 5, this approach gives an approximately “normal” force–velocity relationship (one constant velocity for each load; dashed thick grey line in Fig. 5) despite the force–velocity relationships of the individual half-sarcomeres being anomalous. This “damping” of the anomalous behaviour due to half-sarcomere non-uniformities would be expected to be further enhanced with more half-sarcomeres in series as is the case in studied segments in a muscle fibre where the average sarcomere length of a large number of sarcomeres is controlled and/or measured.

The faithful predictions of the experimental force–velocity relationship, particularly for the 10:1 model means that the power–velocity relationship is also well predicted because power = force × velocity (Fig. S2). However, to give the same absolute power output as in experiments, the force in the 10:1 model (assuming just one myosin binding site per actin target zone) has to be multiplied by ~ 3 to account for the effect of ~ 3 myosin binding sites per target zone in a muscle (Månsson 2019). The ATP turnover rate, as a function of shortening velocity at 0.5 mM Pi, varies between different models (even if the different number of available sites per target zone is taken into account) but there is also appreciable variability among the experimental data (Fig. S2a–c). A general finding, independent of model is that increased [Pi] has negligible effect on the maximum power output per consumed ATP molecule. However because of lower free energy drop per ATP turnover at increased [Pi] the maximum thermodynamic efficiency actually increases with increased [Pi] (Fig. S2d–f).

**Fig. 6:**
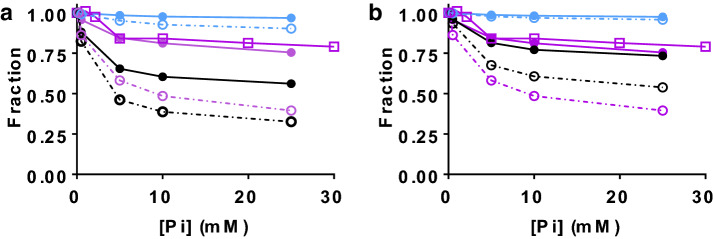
Isometric force, and isometric ATP turnover rate as function of [Pi] for the 10:1 model. Data normalized to value at 0.5 mM Pi. **a** The version of the model most similar to the original one (Rahman et al. 2018, cf. Table S2 for difference). Blue: simulated ATP turnover rate. Black: simulated force. Purple: experimental data for force (circles, Coupland et al. 2001) and ATP turnover rate (open squares, 15 °C, Potma et al. 1995). Filled circles and full lines: 30 °C. Open circles and dashed-dotted lines: 5 °C. Absolute value of simulated ATP turnover rate at 0.5 mM Pi: 2.4 s^−1^ at 30 °C and 0.12 s^−1^ at 5 °C. **b** Model similar to that in a but parameter x_11_ and x_1_ reduced to 7.2 and 6.9 nm, respectively. Colour coding as in **a**. Absolute value of simulated ATP turnover rate at 0.5 mM Pi: 5.5 s^−1^ at 30 °C and 0.36 s^−1^ at 5 °C. (Color figure online)

It is of particular interest to consider changes in isometric force and isometric ATP turnover rate with altered [Pi] because these variables (particularly force) have been studied extensively in experiments. They have also been found in a previous modelling study (Smith 2014) to be useful indices of cross-bridge properties. For the 9:3 model, whether linear or non-linear cross-bridge elasticity is assumed, the ATP turnover rate is increased by increased [Pi]. However, in accordance with recent results (Smith 2014) this effect is reversed if assuming lower affinity between actin and the pre-Pi-release states of actomyosin (Fig. S3). The best predictions for the isometric force and ATP turnover rate are obtained using the 10:1 model for which the predictions are shown in Fig. 6. This model also gives quite faithful reproduction of experimental data for parameter values corresponding to both 5 and 30 °C (Tables S1–S3) and for both sets of the parameter values used in Fig. 4. In all cases, the isometric force was reduced more than the ATP turnover rate by increased [Pi] and the effects of increased [Pi] is predicted to be smaller at high temperature with the quantitatively best reproduction of the data for the alternative set of parameter values (Fig. 6b; same parameter values as in Fig. 4c, d). The predicted isometric ATP turnover rate at 0.5 mM Pi is 2.4 s^−1^ at 30 °C and 0.12 s^−1^ at 5 °C for the version of the model in Fig. 6a whereas these values are increased to 5.5 s^−1^ at 30 °C and 0.36 s^−1^ at 5 °C for the version in Fig. 6b. For a model with 3 sites per target zone, these values would be almost 3-fold higher corresponding to isometric ATPase per head (attached + detached) in the range 6–15 s^−1^ at 30 °C and 0.3–0.7 s^−1^ at 5 °C.

## Discussion

### Choice of models

Recently (Månsson 2019) we found that the present 9:3 model gives quite similar predictions for the maximum shortening velocity under different conditions as well as for the shape of the force–velocity relationship if one, rather than three, myosin binding sites are assumed per actin target zone. However, because the maximum isometric force, the fraction of attached cross-bridges and the ATP turnover rate are better accounted for with three sites than one, a model with three sites (the 9:3 model) is used here.

It is clear from previous work (Månsson 2019) and the present Fig. 3a, b that an important discrepancy between experimental and predicted force–velocity data at 0.5 mM Pi in the 9:3 model with linear cross-bridge elasticity is a lower maximum power output (velocity × force) in the model (see also Fig. S2). This discrepancy is similar if only one site per target zone is assumed (Månsson 2019). Furthermore, the discrepancy is appreciably enhanced if the assumed binding strength in the weak-binding AMDP state is reduced within an experimentally observed range (see Månsson 2016 and references therein), i.e. from a free energy 2.5 k_B_T below that in the MDP state (Fig. 1; tested in Fig. 3) to 0 k_B_T (tested in Fig. S1). The low power-output in the model could imply that critical mechanisms at play in muscle cells are not taken into account. An interesting possibility in this regard is evidence suggesting that the cross-bridge elasticity is non-linear (cf. Kaya and Higuchi 2010; Månsson et al. 2019). If this feature is introduced into the model the discrepancy between the model and experiments is reduced. It is also of interest to note that 20% increase (within experimental uncertainty) in cross-bridge attachment rate [k_on_(x)] without other changes to the 9:3 model would also reduce the difference between the model and experiments (Fig. S1).

However, before setting out to test the effect of altered [Pi] on the force–velocity relationship we also considered the model developed to account for the effects of the small molecular compound blebbistatin (Rahman et al. 2018) because this model (denoted 10:1 model) seems to account very well for the maximum power output of muscle. Furthermore, the fact that it accounts for the blebbistatin effect is of particular interest because the mechanism of action of that compound is tightly associated with the Pi-release mechanism (Kovacs et al. 2004; Rahman et al. 2018). The 10:1 model has not been developed for full incorporation of non-linear cross-bridge elasticity or for three binding sites per target zone. We therefore took the approach here to use it on the assumption of just one binding site per target zone and linear cross-bridge elasticity. This should not pose a severe limitation. First, we recently (Månsson 2019) demonstrated close similarity between 9:1 and 9:3 models provided that the results of the model with just one site is multiplied by ~ 3 with regard to the maximum isometric force, maximum ATP turnover rate and number of attached cross-bridges. Second, there was no appreciable difference in the predicted [Pi] effects on the force–velocity relationship whether using the 9:3 model with linear or non-linear cross-bridge elasticity. As demonstrated recently (Månsson et al. 2019), the effect on the ATP turnover rate varies depending on which cross-bridge states are assumed to exhibit non-linear elasticity. Due to one more state than in the 9:3 model it is outside the scope of this study to consider non-linear elasticity in the 10:1 model. Thus, if the cross-bridge elasticity in muscle is non-linear (presently only convincingly shown for isolated molecules Kaya and Higuchi 2010) it is not clear whether all states would have similar properties (Månsson et al. 2019). A separate analysis of different possibilities in this regard (as previously done for a predecessor of the 9:3 model Månsson et al. 2019) would be needed before including non-linear elasticity into the 10:1 model.

### Strengths and limitations of the models and rationale behind the analysis

Major strengths of all versions of the models tested here are independent origin of model parameter values primarily from biochemical and single molecule studies of actin and myosin, as described by Månsson (2016) and Rahman et al. (2018). Parameter values have further been obtained under conditions as coherent as possible with regard to temperature (30 °C), ionic strength (close to the physiological) and use of myosin from fast mammalian muscle. The key rationale of the analysis (following Månsson 2016, 2019; Månsson et al. 2019; Rahman et al. 2018), is to test whether the bottom-up (from isolated actin and myosin) defined parameter values give good predictions of experimental data from muscle (and other systems with increased complexity). Importantly, no attempts to improve the predictions are made using fitting procedures for further optimizations of parameter values compared to the predefined values. Such efforts would also be of little value considering that the predictions for a range of steady-state contractile variables (force–velocity relationships during shortening, effects of blebbistatin, etc.) are surprisingly good as they are. The latter statement should be viewed in the context of variability in experimental findings between highly renowned labs (e.g. isometric force–[Pi] relationship, Coupland et al. 2001; Tesi et al. 2000; see below for details) and difficulties to find exactly corresponding conditions for the muscle experiments and the biochemical and single molecule experiments where parameter values are obtained. The phenomena accounted for by the models include experimental results on the single molecule level over small motor ensembles in the in vitro motility assay and actomyosin ATPase in solution to contractile performance of living muscle cells (Månsson 2016, 2019; Månsson et al. 2019; Rahman et al. 2018). The latter results include a range of steady state properties such as the force–velocity relationship for shortening, the maximum shortening velocity versus [MgATP] relationship and energetics (e.g. ATP turnover rate and power output) during shortening under different conditions. Additionally, effects of the small molecular substance blebbistatin are well predicted by the 10:1 model (Rahman et al. 2018). The recent modelling studies have focused on steady-state experiments. However, the rate of rise of active isometric force following rapid length changes to zero tension (Månsson 2016) or following rapid perturbations of the Pi-concentration (Rahman et al. 2018) also seem to be accounted for. Furthermore, arguments have also been put forward (Månsson 2019) to suggest that the tension transient in response to fast length steps, with the T1 and T2 relationships (Huxley and Simmons 1971), should be consistent with the model. Eccentric contractions however, seem to involve complexities that require additional assumptions in the models which do not follow directly from experimental findings of isolated proteins (Campbell et al. 2011; Rahman et al. 2018; Rassier and Pavlov 2012).

One may argue that the number of states is too high in the “simple” models to denote these models as “simple”. However, the simplicity lays in the capability to explain data from a range of experimental systems using models without involvement of other protein components than actin and myosin and without other states, transitions and parameter values than those inferred from solution biochemistry or other independent studies, particularly single molecule mechanics. It is of interest to note that the lack of major effects of accessory proteins and emergent phenomena during the shortening part of the force–velocity relationship are consistent with a recent experimental study (Pertici et al. 2018), showing very similar shape of the force–velocity relationship when a small ensemble of myosin motors on a surface interacts with an actin filament in vitro (in the absence of accessory proteins, geometrical order, etc.) as when this relationship is recorded from a living muscle fiber with the fully intact cellular order, accessory proteins, etc. (cf. Månsson 2019; Månsson et al. 1989).

One limitation of the 9:3 model in accounting for the force–velocity data with close to physiological Pi-concentrations (0.5 mM Pi) is an underestimation of the maximum power-output (related to low velocity at intermediate loads). This might imply effects in a real muscle fibre (due to accessory proteins Fujita et al. 2004; Homsher et al. 2003, slippage between sites during shortening Caremani et al. 2013 and mechanosensing effects Marcucci and Reggiani 2016, etc.) that are not accommodated in a model including only actin and myosin and based on a kinetic scheme from solution biochemistry (e.g. Fig. 1). However, interestingly the model would be largely rescued without such assumptions simply by increasing cross-bridge attachment rate by 20% (within experimental uncertainty) and assuming non-linear cross-bridge elasticity. Furthermore, the maximum power-output can be accounted for by the 10:1 model without additional assumptions. The straightforward purpose of the present study is to test whether the simple models, using bottom-up defined parameter values, can also account for the effects of altered [Pi] on muscle contraction without adjustments of the states, transitions and the parameter values. Therefore, as further discussed above, no attempts are made to find alternative parameter values that fit the experimental data better.

The overreaching result is that the models are successful in several regards but new challenges also emerge when the Pi-concentration is varied. These challenges include the anomalous high-force-region of the force–velocity relationship and the increased ATP turnover rate during isometric contraction with increased Pi-concentration (in 9:3 models see further below). However, these deviations must be considered both in relation to experimental complexities and uncertainties and the fact that models are always approximations of the real world. The approximations may fail in certain details while nevertheless satisfactorily representing the overall picture. In this context it is of interest to note the appreciable similarity between the model and experimental data (Caremani et al. 2013) in the overall changes in the force–velocity relationship upon altered [Pi]. Thus, both the model and the experimental data indicate minimal effects of altered [Pi] at shortening velocities above 10% of the maximum velocity but appreciable changes at lower velocities (higher load).

With regard to the increase in the isometric ATP turnover rate with increased [Pi] in the 9:3 model it is important to note that this effect is reversed if the actomyosin affinity is reduced (by reduction in magnitude of either ΔG_on_ or ΔG_w_) as predicted recently using a closely related model (cf. Smith 2014). However, this effect comes at the price of a markedly reduced power output at 0.5 mM Pi. We therefore favour the 10:1 model because, in terms of this model, the isometric ATP turnover rate tends to be reduced with increased [Pi] for parameter values that also account for the high power output. With regard to other effects of altered [Pi] on energetics, such as the ATP turnover rate during shortening and efficiency, these effects are small (Fig. S2) as are those on the force–velocity relationship at all velocities other than the lowest ones.

### Relation to experimental complexities and uncertainties: suggestions for new experiments

The existence of uncertainties and complexities in experimental data for contractile variables has been demonstrated in a range of previous studies and include: (1) lack of complete experimental data and/or lack of data obtained under coherent conditions of e.g. temperature, ionic strength, myosin isoform, species, etc., (2) variability between data obtained in different experimental systems such as in vitro motility assays, myofibrils and muscle fibres, (3) uncertainties in the interpretation of experimental data e.g. due to filament-elasticity whose properties are not yet fully characterized (Fusi et al. 2010, 2014; Månsson 2010b; Månsson et al. 2018; Offer and Ranatunga 2010) or poorly understood underlying processes (e.g. T2-curves for stretch; Offer and Ranatunga 2016) and (4) effects of emergent properties due to the organization of a muscle fibre in sarcomeres where there are differences in the half-sarcomere properties (Campbell et al. 2011; Minozzo et al. 2013). Limited number of data points in the high-force region of the force–velocity relation (velocity < ~ 10% V_0_), as is often seen (e.g. Caremani et al. 2013), belongs to the first class of uncertainties as does the lack of data (to the best of my knowledge) for the relationship between [Pi] and isometric ATPase at 30 °C. Somewhat different effects of [Pi] on isometric force at a given temperature in different labs (Coupland et al. 2001; Tesi et al. 2000), fall into the second class, with different types of complexities in experiments using skinned muscle fibres (Coupland et al. 2001) and myofibrils (Tesi et al. 2000). Further, the different effects of altered [Pi] on cross-bridge stiffness and the number of attached cross-bridges suggested by different muscle mechanics studies belong to the third class as the characteristics of the myofilament elasticity are central in the interpretation of stiffness data. Due to this fact, results based on stiffness measurements are not explicitly considered here. Finally, the disappearance or emergence of contractile phenomena, compared to the single filament level, in studies of interconnected half-sarcomeres (Campbell et al. 2011; Edman and Reggiani 1984a, b; Gordon et al. 1966; Rassier and Pavlov 2012; Vilfan and Duke 2003) belongs to the fourth class. In the latter connection the results in Fig. 5 are of particular relevance for the present study, suggesting the possibility that an anomalous force–velocity relationship at high loads may go undetected in studies on muscle cells where many sarcomeres in parallel and in series are studied.

In Figs. 3 and 4 model simulations are only compared to experimental data obtained under low [Pi] conditions corresponding to unfatigued conditions in a muscle cell. There are different reasons for this. Whereas detailed force–velocity data for intact muscle exist under fatiguing conditions (Curtin and Edman 1994), such data are complex due to other simultaneous intracellular changes during fatigue (Debold 2012) and the exact [Pi] level is not known. For skinned muscle fibres, on the other hand, where the myofibrillar [Pi] concentration can be directly controlled, the amount of available data is, to the best of my knowledge, limited. This both applies to the range of different [Pi] concentrations tested and to the availability of data for closely spaced loads/velocities in the high-load, low-velocity range (cf. Caremani et al. 2013). For testing the predictions of the current models at varied [Pi] it would clearly be of great interest with more detailed studies of the force–velocity relationship in the low-velocity region and for a range of [Pi] levels.

With regard to an anomalous force velocity relationship such a property may be associated with mechanical oscillations at loads close to the isometric force as shown previously (e.g. Julicher and Prost 1995; Vilfan and Frey 2005; Vilfan et al. 1999). It would therefore be of interest if tendencies for oscillatory behaviour are enhanced in muscle or myofibril experiments upon increasing the phosphate concentration. An effect that is interesting to mention in this context is the enhancement in fatiguing conditions (associated with both reduced pH and increased [Pi] Debold 2012) in a living muscle fibre of rapid length oscillations following a rapid change in load close to the isometric force (Edman and Curtin 2001). Whereas the effect was mimicked by intracellular acidification (Edman and Curtin 2001) it would be of interest to repeat the study under conditions of constant pH and increased [Pi] using skinned muscle fibres or myofibrils where the effects of acidification and increased phosphate concentration may be isolated from each other. Further experimental studies that would be of great interest would be evaluation of the force–velocity relationship of individual half-sarcomeres in myofibrils at different [Pi], using a half-sarcomere myofibril mechanics set-up similar to that described previously (Minozzo et al. 2013). The present model would be corroborated if such experiments demonstrate enhancements of an anomalous force–velocity relationships at increased [Pi] compared to Pi concentrations around 1 mM. An alternative approach to test the model may be use of isolated proteins, i.e. a force–velocity assay for small ensembles of myosin motors (Pertici et al. 2018).

In addition to uncertainties in the experimental data, to which the model predictions are compared, there are also uncertainties in model parameter values obtained in independent experiments. In the present models these uncertainties relate to (1) the attachment range of a cross-bridge (e.g. appreciably lower in the 10:1 model than in the 9:3 model), (2) the cross-bridge stiffness at positive x-values (1.7–3 pN/nm) (Månsson et al. 2018) and whether the cross-bridge elasticity is linear or non-linear (Månsson et al. 2019) and (3) the absolute magnitude of the power-stroke distance (~ 7 to ~ 10 nm) (Månsson et al. 2018). It is shown in Fig. 4c, d that small changes in these parameter values within the experimental uncertainties could alter model predictions to better conform with experimental data (Rahman et al. 2018).

### More complex models: to be or not to be?

It is well known that almost anything can be explained by models with sufficiently large number of states and transitions (Mayer et al. 2010). It is therefore essential that introduction of new states and transitions into models rest on a firm ground with independent support, preferably in several types of studies independent from those where the model is used to derive predictions. These characteristics largely apply to the models used here as laid out above and in greater detail elsewhere.

At the present stage, recent suggestions about the importance of branched pathways at increased [Pi] (Debold et al. 2013), slippage between sites (Caremani et al. 2013) and/or loose coupling between biochemical and mechanical states (Caremani et al. 2013) cannot be excluded. However, in view of the uncertainties and variabilities of the experimental data as well as the success of the simple models in accounting for a range of experimental findings, I presently see no reason to abandon these simple models. With their independent support from solution biochemistry, single molecule studies and ultrastructural data they also have a good potential to provide understanding of processes on the molecular scale on basis of wide variety of experimental studies.

## Conclusions

It is shown above that models assuming unbranched pathways, tight coupling between force-generation and biochemical transitions and no slippage of cross-bridges between sites, account quite well for a range of experimental data at varied [Pi]. Particularly successful in this regard is the 10:1 model similar to that of Rahman et al. (2018), found to account for a range of blebbistatin effects in addition to data obtained under physiological conditions. A challenge for all models tested is the anomalous force–velocity relationship predicted to appear at high loads (low velocity) when [Pi] is increased. This effect may be counteracted in the models by small changes (within experimental uncertainties) of the parameter values. However, as a main idea emerging from this study, it is important to consider the possibility that the anomalous force–velocity relationship is actually a real property of individual half-sarcomeres at high [Pi] despite going undetected in experimental studies on muscle cells. Experimental tests of this idea are proposed.

## Electronic supplementary material

Below is the link to the electronic supplementary material.

Supplementary material 1 (DOCX 1602 kb)

## Footnotes

1 Taken from the literature value (~ 400 kPa) assuming that: (1) 80% of the fibre cross-section is taken up by myofibrils, (2) there are 294 myosin heads per half-thick filament, (3) the two heads of a given myosin molecule are independent and (4) the area of the hexagon with a myosin filament in the center and thin actin-containing filaments in the corners is 1.6 × 10^−15^ m^2^.

2 Similar calculation as in footnote 1 but instead taking the force per cross-sectional area (150 kPa) from Månsson et al. (1989).
