# Supplementary material for: The effects of inorganic phosphate on muscle force development and energetics: challenges in modelling related to experimental uncertainties
Source: J Muscle Res Cell Motil. 2019 Oct 16;42(1):33–46. doi: 10.1007/s10974-019-09558-2 (PMC7932973; doi:10.1007/s10974-019-09558-2)
Supplement: Supplementary file 1 — Supplementary material 1 (DOCX 1602 kb) [file 10974_2019_9558_MOESM1_ESM.docx]

**Supporting Information**

**The effects of inorganic phosphate on muscle force development and energetics – challenges in modelling related to experimental uncertainties**

by

Alf Månsson

*Models*

Two slightly different models are used, both developed from that in ([Mansson 2016](#_ENREF_10)). The model 9:3 ([Mansson 2019](#_ENREF_11)) is characterized by 9 biochemical/structural states and 3 myosin binding sites per target zone at 36 nm spacing along the actin filament (main Fig. 1a, b, c). Details in ([Mansson et al. 2019](#_ENREF_12))). The other model, with 10 states and 1 binding site per actin target zone (main Fig. 1a, d, e), is slightly modified (cf. Table S2) from that in ([Rahman et al. 2018](#_ENREF_14)). As motivated previously (cf. ([Hill 1974](#_ENREF_5); [Huxley 1957](#_ENREF_6); [Mansson 2019](#_ENREF_11))) a uniform distance (x) distribution of myosin-heads to the center of of the nearest actin target zone is assumed as well as independent interaction of the two heads of each myosin molecule with actin.

The model states and their properties are defined in the main Fig. 1. The different myosin (M) and actomyosin (AM) states have either substrate (ATP; T) or products (ADP, D; inorganic phosphate, P or Pi) at the active site. Both models encompass three states with myosin detached from actin or weakly attached (MT, MDP, AMDP; cf. Fig. 1). The attached states are of different biochemical and structural types as indicated in Fig. 1. The subscripts “L” and “H” in AMDL and AMDH in main Fig. 1 refer to “low” and “high” force, respectively corresponding to the state before and after the main force-generating transition, respectively ([Huxley and Simmons 1971](#_ENREF_7)). The stiffness in the AMDP state is set to zero ([Rahman et al. 2018](#_ENREF_14)).

**Model 9:3**

In this section, elastic properties and rate functions are given for the central site in the target zone. The corresponding functions for the peripheral sites are obtained by exchanging xi (x1, x2, x3; in nm) for xi+5.5 and xi-5.5, respectively. Parameter values are given in Tables S1-S2. The cross-bridge stiffness in the different states, AMDL (ksi(x)), AMDH (ksii(x)) and AM (ksiii(x); lumped together with AMD; AM/AMD) is constant (2.8 pN/nm) with linear cross-bridge elasticity. If the cross-bridge elasticity is assumed non-linear, the stiffness varies as follows:

ksi(x)=2.8 pN/nm for x≥x1 (1a)

ksi(x)=0.12 pN/nm for x<x1 (1b)

ksii(x)=2.8 pN/nm for x≥x2 (2a)

ksii(x)=0.12 pN/nm for x<x2 (2b)

ksiii(x)=2.8 pN/nm for x≥x3 (3a)

ksiii(x)=0.308+c(2.8-0.308) pN/nm for -4 nm ≤x< x3 (3b)

ksiii(x)=0.0326+ c(2.8-0.0326) pN/nm for -75 nm ≤x- x3 <-4 nm (3c)

ksiii(x)=2.8 pN/nm for -90 nm≤ x-x3<-75 nm(3d)

ksiii(x)=0 for x-x3 <-90 nm (with obligate cross-bridge detachment) (3e)

The parameter c in Eqs. 3b-3c was set to 1 and 0.0255, to simulate linear and non-linear cross-bridge elasticity, respectively.

The equilibrium constant, Kw for weak myosin head binding (Fig. 1C) is given by Kw=exp(ΔGw) inside the range: –2.8 nm<x<18.2 nm and 0 outside this range where ΔGw denotes the free energy difference between the MDP state and the AMDP state. Below, any free energy differences (ΔGw, $\Delta$GAMDP-AMDL, $\Delta$GAMDL-AMDH and $\Delta$GAMDH-AM) are given in units of kBT (≈ 4 pN nm) where kB is the Bolzmann constant and T is the absolute temperature.

The rate function for the transition from the AMDP to the AMDL state (Fig. 1) is given by:

kon(x)=kon´exp[$\Delta$GAMDP-AMDL–(ksi(x)/2)(x-x1)2/(2kBT)]

for -2.8 nm<x<18.2 nm and 0 otherwise. (4)

The reversal of this transition is governed by the rate function:

kon-rev(x)=kb(x)[Pi]/(KC+[Pi]) (5)

where [Pi] is the concentration of inorganic phosphate, KC is the phosphate dissociation constant and:

kb(x)= kon´exp[(ksi(x)/2)(x-x1)2/(2kBT)] (6)

The power-stroke ([Huxley and Simmons 1971](#_ENREF_7)) is assumed to be a rapid equilibrium with equilibrium constant given by:

KLH(x)=kLH+(x)/kLH-(x) (7)

Here,

kLH+(x)=kLH-(x) exp($\Delta$GAMDL-AMDH +ksi(x)(x-x1)2/(2kBT)-ksii(x)(x-x2)2/(2kBT)) (8)

and

kLH-(x)=2000 s-1 (9)

The strain dependent transition from the AMDH to the AMD state ([Albet-Torres et al. 2009](#_ENREF_1); [Capitanio et al. 2006](#_ENREF_3); [Nyitrai and Geeves 2004](#_ENREF_13)) is governed by:

$k_{5}\left( x \right)=k_{5}\left( x_{1} \right)$ exp($\Delta$GAMDH-AM+ksii(x)(x-x2)2/(2kBT)-GAM(x))) (10a)

where

/ kBT (10b)

with being a force function with constant slope (2.8 pN/nm) in the case of linear cross-bridge elasticity or approximating the force-extension relation for x<0 nm derived in ([Kaya and Higuchi 2010](#_ENREF_8)) in the case of non-linear elasticity.

The detachment rate function from the AMD to the MT state is approximated by:

koff(x)$=\frac{k_{2}(x)k_{6}\left[ MgATP \right]}{\frac{k_{6}}{K_{1}}+(k_{2}(x)+k_{6})\left[ MgATP \right]}=\frac{k_{2}(x)\left[ MgATP \right]}{\frac{1}{K_{1}}+\frac{k_{2}(x)}{k_{6}}\left[ MgATP \right]+[MgATP]}$ (11)

where

$k_{2}\left( x \right)=k_{2}(0)exp \left( \frac{\left| F_{AM}(x-x_{3}) \right|{\cdot x}_{crit}}{k_{B}T} \right)$ (12)

Here, k2(0) and k6 are rate constants for ATP induced detachment from the AMT state at x=0 and ADP-dissociation from the AMD state, respectively. The parameter K1 is the equilibrium constant for MgATP binding to the AM/AMD state (Fig. 1b). Finally, xcrit is a parameter ([Bell 1978](#_ENREF_2)) defining strain-sensitivity of k2(x). Because, it is assumed that [MgADP] ≈ 0 mM, the transition from the AMDH to the AM/AMD state is assumed to be irreversible.

The differential equations for the state probabilities (for all l,k) were solved for steady-state contraction with different constant velocities, v:

$\frac{da_{l}}{dx}=(\sum_{k}^{n1} k_{kl}\left( x \right)a_{k}\left( x \right)-\sum_{k}^{n2} k_{lk}{(x)a}_{l(x)})/v$ (13)

Here, ak(x) and al(x) are state probabilities for the states (Fig. 1): MT (l,k=4), MDP (l,k =5), AMDP (l,k=6), AMDL (l,k =1,7,8), AMDH (l,k=2,9,10) and AM/AMD (l,k=3,11,12). The MT, MDP and AMDP states are shared between all three sites in a target zone whereas there are three different attached states for each site in the target zone. The rate functions kkl(x) and klk(x) govern transitions into state l from n1 neighboring states and out of state linto n2 neighboring states, respectively. The model simulations were implemented by numeric solution of the master equations (Eq. 13) followed by calculation of force (<F>), number of attached cross-bridges (<Na>) and ATP turnover rate (<ATPase>) as the appropriate averages over all available myosin heads:

$<F>= \frac{\sum_{k=1}^{12} \int_{-50}^{19.5} k_{s}^{k}\left( x \right)a_{k}\left( x \right)(x-x_{k})dx}{36}$ (14)

$<Na>= \frac{\sum_{k=1 (only attached)}^{12} \int_{-50}^{19.5} a_{k}\left( x \right)dx}{36}$ (15)

$<ATPase>= \sum_{k=3,11,12} \int_{-50}^{19.5} k_{off}a_{k}(x)dx/36$ (16)

where ksk(x) is the cross-bridge stiffness (cf. Eqs. 1a-3e). The quantities xk are given in Table S1 for the central site in the target zone (k=1,2,3; see above) and for the peripheral sites by subtraction or addition of 5.5 nm from/to the xk-values.

**Model 10:1**

Model 10:1 was implemented on the assumption of linear cross-bridge elasticity (ks=2.8 pN/nm). The majority of the rate functions are similar to those given in Eqs. 7-12 for the central site in Model 9:3 with minor differences in parameter values (Tables S1-S2). Changes in parameter values to simulate contractile properties at low temperature are given in Table S3. The following rate functions are needed in addition to those in model 9:3 to accommodate the extra states in Model 10:1 as follows (Eqs. 17-20):

The transition from the weakly and non-stereospecifically bound AMDP state to pre-power stroke state AMDPPP state is governed by the rate function:

kon(x) = kon´ exp($\Delta$Gon - ks (x-x1)2/ kBT) (17)

where $\Delta$Gon is the difference between the free energy minima of the AMDP and AMDPPP states.

The reversal of this process is governed by:

kon-rev(x) = kon´ exp(ks (x-x1)2/kBT) (18)

Next, following ([Llinas et al. 2015](#_ENREF_9)) and as formalized in ([Rahman et al. 2018](#_ENREF_14)), a transition occurs into a Pi-release state (AMADPPiR) from which Pi is rapidly and reversibly released, with transition into the AMDL state. Denoting the difference between the minima for the free energies in the AMDPPP and the AMDPPiR states as $\Delta$GPiR, the relevant rate functions for the transitions back and forth between the AMDPPP and the AMDL state are given by:

kP+(x)= kP+´exp($\Delta$GPiR/2 -(ks/2)(x-x1)2/ (2kBT) +(ks/2)(x-xw)2/(2kBT))) (19)

kp-(x)=kP+´[Pi]/([Pi]+Kp) exp ($\Delta$GPiR)/ /2+(ks/2)(x-x1)2/(2kBT)-(ks/2)(x-xw)2/(2kBT)) (20)

Force, number of attached cross-bridges and the ATP turnover rate are then calculated along the same principles as for model 9:3 (Eqs. 14-16).

**Implementation of models**

The detailed approach for numerical integration has been motivated previously as well as the basis for the wide integration range in Eqs. 14-16 ([-50, 19.5] nm) ([Mansson et al. 2019](#_ENREF_12)) in relation to the narrower, 36 nm, physical separation of neighboring target zones.

Differential equations were solved numerically using a Runge-Kutta Fehlberg (4/5) integration algorithm in Simnon (Department of Automatic Control, Lund Institute of Technology, Sweden). The source code was given in the Supplementary Material of ([Mansson 2019](#_ENREF_11)) with explanations and comments. For stability in the numerical computations, the value of any rate function (Eqs (4-12; 17-20)) was limited to between a minimum (rmin) of 1 x 10-6 s-1­ and a maximum (rmax) of 100 000 s-1 for isometric contraction or 1000 000 s-1 for the fastest velocities of shortening. If any of the limits was exceeded the parameter value was set to either rmax or rmin.

**Supporting Figures**

**Fig. S1. Force-velocity relationships simulated using model 9:3 with alternative parameter values at 0.5 mM Pi. a.** Linear cross-bridge elasticity. Data obtained with standard model parameter values (Tables S1-S2) in main Fig. 3a are shown together with experimental data (purple) from main Fig. 3. In addition, simulated data are shown on the assumption of lower actomyosin affinity in the AMDP state (Kw=1 instead of 12; ΔGw = 0 instead of -2.5 kB T) or 20 % higher value of the rate limiting constant, kon, for cross-bridge attachment. **b.** Data from a in the high-force, low-velocity ranges. **c.** Non-linear cross-bridge elasticity. Same symbols and colour coding as in a. **d**. Data from c in the high-force, low-velocity regions.

**Fig. S2. Detailed energetics of contraction for different models at 0.5 and 25 mM Pi and shortening at different velocities. a.** Simulation of ATP turnover rate (black circles) vs velocity using model 9:3 with linear cross-bridge elasticity. Filled symbols: 0.5 mM Pi; Open symbols: 25 mM Pi. Purple symbols: Experimental data scaled to 30 oC as explained in ([Mansson et al. 2019](#_ENREF_12)). Purple line: ([He et al. 1999](#_ENREF_4)). Purple squares: ([Sun et al. 2001](#_ENREF_16)). Purple triangles: ([Reggiani et al. 1997](#_ENREF_15)). **b.** Simulation of ATP turnover rate (black circles) vs velocity using model 9:3 with non-linear cross-bridge elasticity. Experimental data plotted using same symbols and colours as in a. **c.** Simulation of ATP turnover rate vs velocity using model 10:1 with linear cross-bridge elasticity. Similar colour and symbol coding as in a. Simulated data multiplied by 3. **d.** Power output (black, purple; left vertical axis) and thermodynamic efficiency (red; right vertical axis) vs velocity for 9:3 model assuming linear cross-bridge elasticity. Black symbols: Simulated power output. Purple symbols: Power output from Månsson et al. (1989). Red symbol: Simulated efficiency. Filled symbols: 0.5 mM Pi. Open symbols: 25 mM Pi. **e.** Same type of data as in d (and same symbol coding) but using model 9:3 assuming non-linear cross-bridge elasticity. **f**. Same type of data as in d (and same symbol coding) but using model 10:1 with linear cross-bridge elasticity. Simulated power output multiplied by 3 to account for 3 binding sites per actin target zone.

**Fig. S3. The ratio between isometric ATP turnover rate at 25 mM and 0.5 mM Pi as function of the free energy of binding** **in the weak-binding AMDP state in the 9:3 model**. Note that the lower value of ΔGweak means higher affinity suggesting that the isometric ATP turnover rate is increased by increased [Pi] at high affinity in the AMDP state but reduced by increased [Pi] at low affinity (cf. Smith, 2014).

**Supporting Tables**

**Table S1**. Parameter valuesa determining shape of free energy diagrams for simulation of contractile properties of fast mammalian muscle at 30 oC

| **Parameter** | **Model 9:3 Numerical value used, linear and (non-linear) elasticity** | **Model 10:1 Numerical value used, linear elasticity** |
| --- | --- | --- |
| x11 (AMDP, AMDPPP) | NA | 8.7 nm |
| x1 (AMDPPiR, AMDL) | 7.7 nm | 7.7 nm |
| x2 (AMDH) | 1.0 nm | 1.0 nm |
| x3 | 0 nm | 0 nm |
| $\Delta$Gw(MDP- AMDP) | 2.5 kBT | 0 kBT |
| $\Delta$GAMDP-AMDP-PP ≡$\Delta$Gon (AMDP–AMDPPP) | NA | 0.7 kBT |
| $\Delta$GPiR (AMDPPP –AMDPPiR) | NA | 1 kBT |
| $\Delta$GAMDP-PiR-AMDP-L (AMDPPiR –AMDL) | NA | kBT ln([Pi]/KC) |
| $\Delta$GAMDP-AMDL (AMDP–AMDL) | 0.7 kBT + kBT ln([Pi]/KC) | NA |
| $\Delta$GAMDL-  AMDH (AMDL- AMDH) | 15 kBT | 14 kBT |
| $\Delta$GAMDH-  AMD  (AMDH- AMD) | 2 kBT | 2 kBT |
| $\Delta G_{ATP}$ | 13.1 + ln ([MgATP]/ ([MgADP][Pi]) kBT | 13.1 + ln ([MgATP]/ ([MgADP][Pi]) kBT |
| Ks | 2.8 (0.03 -2.8) pN/nm | 2.8 pN/nm |

Footnotes to Table S1

NA: Not applicable; IS: ionic strength

a The parameter values were from two-headed myosin motor fragments from fast skeletal muscle of rabbit at 30oC, ionic strength 130-200 mM, pH 7-8 unless otherwise stated. For further details, see ([Mansson 2019](#_ENREF_11); [Mansson et al. 2019](#_ENREF_12); [Rahman et al. 2018](#_ENREF_14))

**Table S2**. Parameter valuesa defining rate functions and kinetic constants for simulation of contractile properties of fast mammalian muscle at 30 oC.

| **Parameter** | **Model 9:3 Numerical value used, linear and (non-linear) elasticity** | **Model 10:1 Numerical value used, linear elasticity** |
| --- | --- | --- |
| k+3 + k-3 | 220 s-1 | 220 s-1 |
| K3 | 10 | 10 |
| k-5 | 2000 s-1 | 2000 s-1 |
| Kc | 10 mM |  |
| kon´ | 100 s-1 | 130 s-1 |
| kP+´ | NA | 3000 s-1 b |
| xcrit | 0.6 nm (0 nm) | 0.6 nm |
| k6 | 5000 s-1 | 5000 s-1 |
| k-6 | 14 290 mM-1 s-1 | 14 290 mM-1 s-1 |
| Physiological [Pi] | 0.5 mM | 0.5 mM |
| K1 | 1.7 mM-1 | 1.7 mM-1 |
| k2 | 1800 s-1 | 2000 s-1 |

a The parameter values were from two-headed myosin motor fragments from fast skeletal muscle of rabbit at 30oC, ionic strength 130-200 mM, pH 7-8 unless otherwise stated. For further details, see ([Mansson 2019](#_ENREF_11); [Mansson et al. 2019](#_ENREF_12); [Rahman et al. 2018](#_ENREF_14)).

b Note, difference from model in ([Rahman et al. 2018](#_ENREF_14)) where the same parameter value was set to 1000 s-1 under control conditions. Here, a higher value was found necessary in order to achieve the experimentally observed maximum shortening velocity without changing other parameter values from their literature data.

**Table S3**. Changes in parameter valuesa for simulation of contractile properties of fast mammalian muscle at 5 oC using model 10:1

| **Parameter** | **30oC** | **5oC** |
| --- | --- | --- |
| $\Delta$GAM*DL-  AM´DH ≡ $\Delta$**GLH**  (AM*DL- AM*DH) | 14 kBT | 6 kBT |
| k+3 + k-3  (Recovery stroke+hydrolysis) | 220 s-1 | 12.5 s-1 |
| K3 | 10 | 4 |
| kon´ | 130 s-1 | 25 s-1 |
| k2 | 2000 s-1 | 413 s-1 |
| kP+´ | 3000 s-1 | 250 s-1 |

aSame parameter values as in Tables S1-S2. Parameter values not given here are assumed identical to those given in Tables S1-S2

**Supporting References**

Albet-Torres N et al. (2009) Drug effect unveils inter-head cooperativity and strain-dependent ADP release in fast skeletal actomyosin J Biol Chem 284:22926–22937

Bell GI (1978) Models for the specific adhesion of cells to cells Science 200:618-627.

Capitanio M et al. (2006) Two independent mechanical events in the interaction cycle of skeletal muscle myosin with actin Proc Natl Acad Sci U S A 103:87-92

He ZH, Chillingworth RK, Brune M, Corrie JE, Webb MR, Ferenczi MA (1999) The efficiency of contraction in rabbit skeletal muscle fibres, determined from the rate of release of inorganic phosphate J Physiol (Lond) 517:839-854

Hill TL (1974) Theoretical formalism for the sliding filament model of contraction of striated muscle. Part I Prog Biophys Mol Biol 28:267-340

Huxley AF (1957) Muscle structure and theories of contraction Prog Biophys Biophys Chem 7:255-318

Huxley AF, Simmons RM (1971) Proposed mechanism of force generation in striated muscle Nature 233:533-538

Kaya M, Higuchi H (2010) Nonlinear elasticity and an 8-nm working stroke of single myosin molecules in myofilaments Science 329:686-689

Llinas P et al. (2015) How actin initiates the motor activity of Myosin Dev Cell 33:401-412

Mansson A (2016) Actomyosin based contraction: one mechanokinetic model from single molecules to muscle? J Muscle Res Cell Motil 37:181-194

Mansson A (2019) Comparing models with one versus multiple myosin-binding sites per actin target zone: The power of simplicity J Gen Physiol 151:578-592

Mansson A, Persson M, Shalabi N, Rassier DE (2019) Non-linear actomyosin elasticity in muscle? Biophys J 116:330–346

Nyitrai M, Geeves MA (2004) Adenosine diphosphate and strain sensitivity in myosin motors Philos Trans R Soc Lond B Biol Sci 359:1867-1877

Rahman MA, Usaj M, Rassier DE, Mansson A (2018) Blebbistatin Effects Expose Hidden Secrets in the Force-Generating Cycle of Actin and Myosin Biophys J 115:386-397

Reggiani C, Potma EJ, Bottinelli R, Canepari M, Pellegrino MA, Stienen GJ (1997) Chemo-mechanical energy transduction in relation to myosin isoform composition in skeletal muscle fibres of the rat J Physiol 502:449-460.

Sun YB, Hilber K, Irving M (2001) Effect of active shortening on the rate of ATP utilisation by rabbit psoas muscle fibres J Physiol 531:781-791.
